# Case report: A case of high grade serous carcinoma of peritoneal origin

**DOI:** 10.3389/fonc.2024.1323650

**Published:** 2024-03-25

**Authors:** Yiming Liu, Sixian Wang, Yingchao Wu, Guowei Chen, Junling Zhang, Xin Wang

**Affiliations:** First Hospital, Peking University, Beijing, China

**Keywords:** Peritoneal carcinoma, hematemesis, obstruction, emergency exploratory laparotomy, dignosis

## Abstract

This case report describes an 80-year-old female patient admitted to the emergency department due to abdominal distension, abdominal pain, and hematemesis persisting for three days. Subsequent postoperative pathological examination confirmed the diagnosis of peritoneal cancer. The occurrence, diagnosis, treatment, and prognosis of primary peritoneal cancer (PPC) are presented in detail. PPC is a type of cancer originating from the primary peritoneal mesothelium organization, causing diffuse malignant tumors in the abdominal and pelvic regions. Due to the lack of specific clinical manifestations for this disease, the importance of early diagnosis and treatment is hereby emphasized. The article also mentions the histological source of this type of cancer and the advantages of preoperative intraperitoneal chemotherapy in improving the efficacy of PPC treatment. Finally, the importance of a comprehensive treatment approach and proficient use of targeted therapy techniques are highlighted to enhance the treatment outcomes of PPC.

## Introduction

Peritoneal carcinoma encompasses malignant tumors that originate and/or progress within the peritoneum. This classification includes both primary and secondary forms (1). Primary peritoneal carcinoma (PPC) and malignant peritoneal mesothelioma are notable examples of the former, while peritoneal metastasis originating from various tumors, including gastrointestinal and gynecological tumors, characterizes the latter (2). The clinical manifestations of peritoneal carcinoma often lack specificity, making it a research hotspot to understand its occurrence, development, clinical diagnosis, treatment, and prognosis. In our clinical work, there involved encountered a patient with peritoneal carcinoma presenting primarily with abdominal pain and hematemesis.

**Figure 1:**
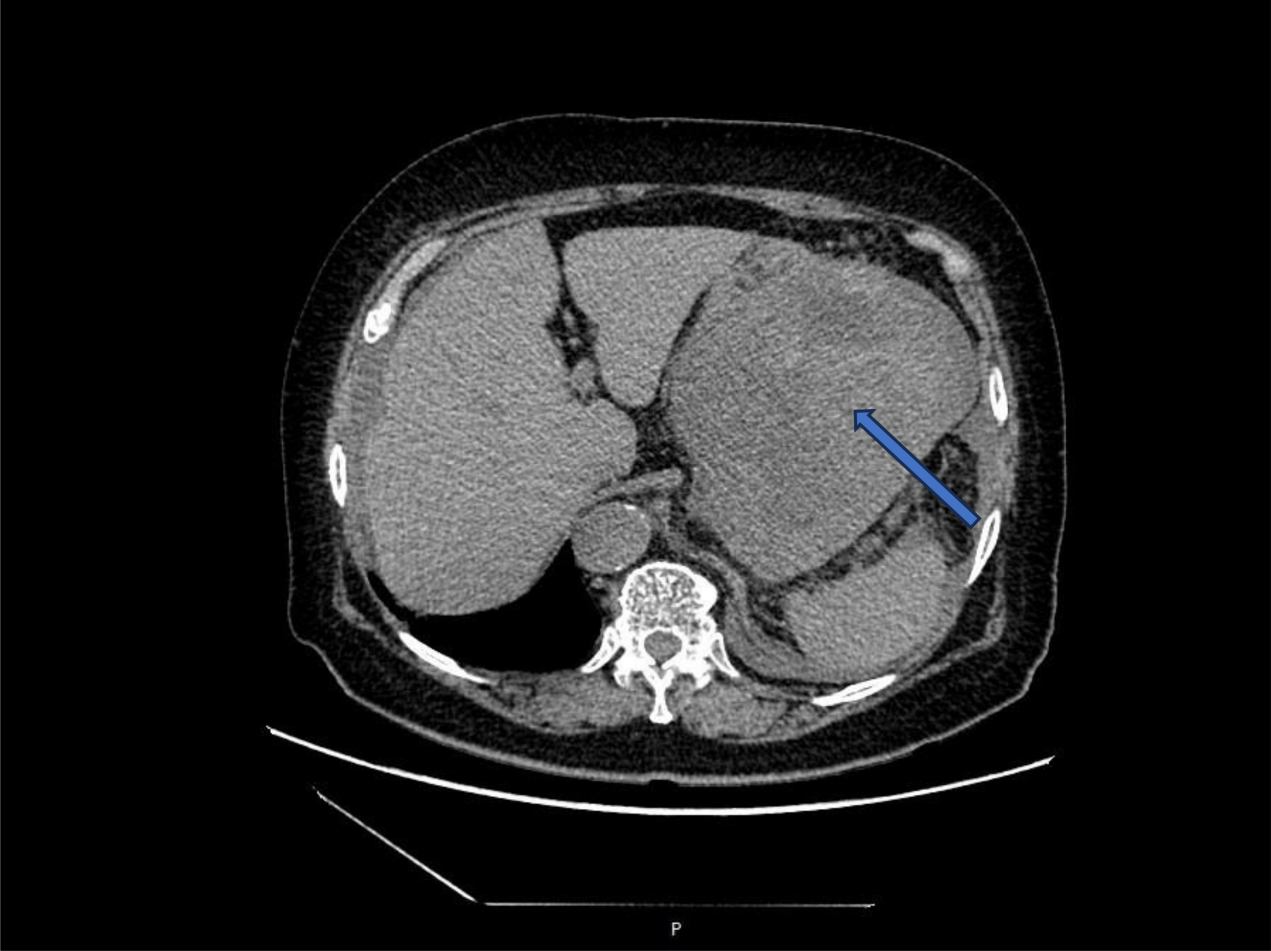
CT of midsection, with the marked areas representing lesions.

**Figure 2:**
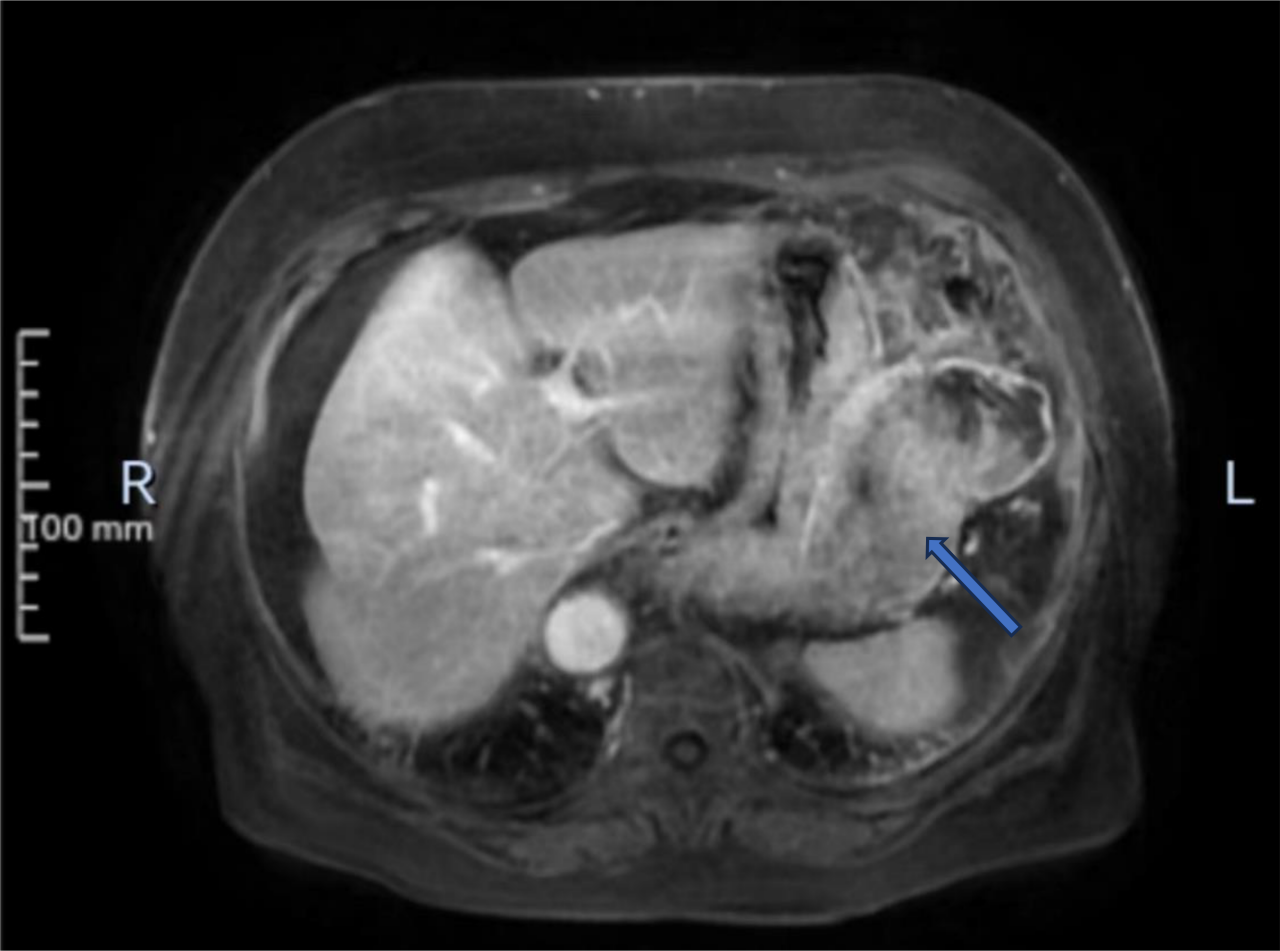
MRI of midsection, with the marked areas representing lesions.

An 80-year-old female patient was admitted to the hospital as an emergency case due to abdominal distension, abdominal pain, and hematemesis persisting for three days. The patient had stopped defecating for more than a week and had a hemoglobin level of 60g/L upon arrival at the external hospital. On admission, the patient presented with total abdominal distension, dull pain in the left upper abdomen, while no obvious signs of peritoneal irritation were observed. The ultrasonography indicated mixed echo in the left upper abdomen with abdominal fluid accumulation. CT scans (**Figure 1**) revealed a mass in the left abdominal cavity with a high probability of gastrointestinal stromal tumor. The MRI impression (**Figure 2**) indicated a high likelihood of a malignant space-occupying lesion on the left side of the abdominal cavity. The patient suffered from intestinal obstruction for more than a week and hematemesis for three days, with a six-year history of hypertension, and the highest recorded blood pressure reached 220mmHg. Daily oral administration of Bioxitone has been effective in managing the blood pressure. He had been experiencing bilateral knee pain for over two decades, which could be alleviated with oral analgesics, and had no other notable medical history.

An emergency exploratory laparotomy was performed immediately after admission. During the operation, the surgeons discovered about 800ml of non-coagulated blood in the abdominal cavity. The tumor, measuring 15cm in diameter, was detected in the greater curvature of the stomach and the lesser omental sac behind the stomach. The tumor envelope was smooth, broken and bleeding, and there were multiple adhesions to the surrounding area. The surgeons excised multiple metastatic nodules on the greater omentum.

**Figure 3:**
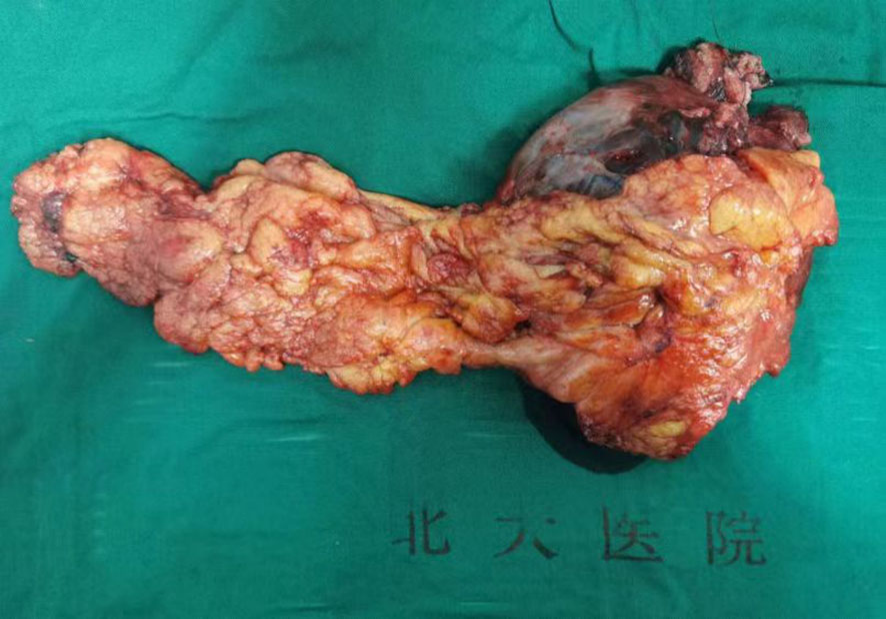
Post-tumor resection specimen.

The tumor involved the adjacent gastric wall and reached the muscular layer. The anterior wall of the mass involved part of the posterior wall of the transverse colon and impinged on the left abdominal wall and the left diaphragm, while the posterior wall impinged on part of the caudal capsule of the pancreas. Part of the gastric wall and part of the tail pancreatic tissue were removed, and the affected colonic serous membrane was excised and restored using absorbable silk suture (**Figure 3**). The operation lasted for approximately three hours with minimal intraoperative bleeding of around 50ml. Considering the significant hematemesis experienced by the patient prior to the surgery, an intraoperative infusion of 800ml of red blood cell suspension was administered.

**Figure 4:**
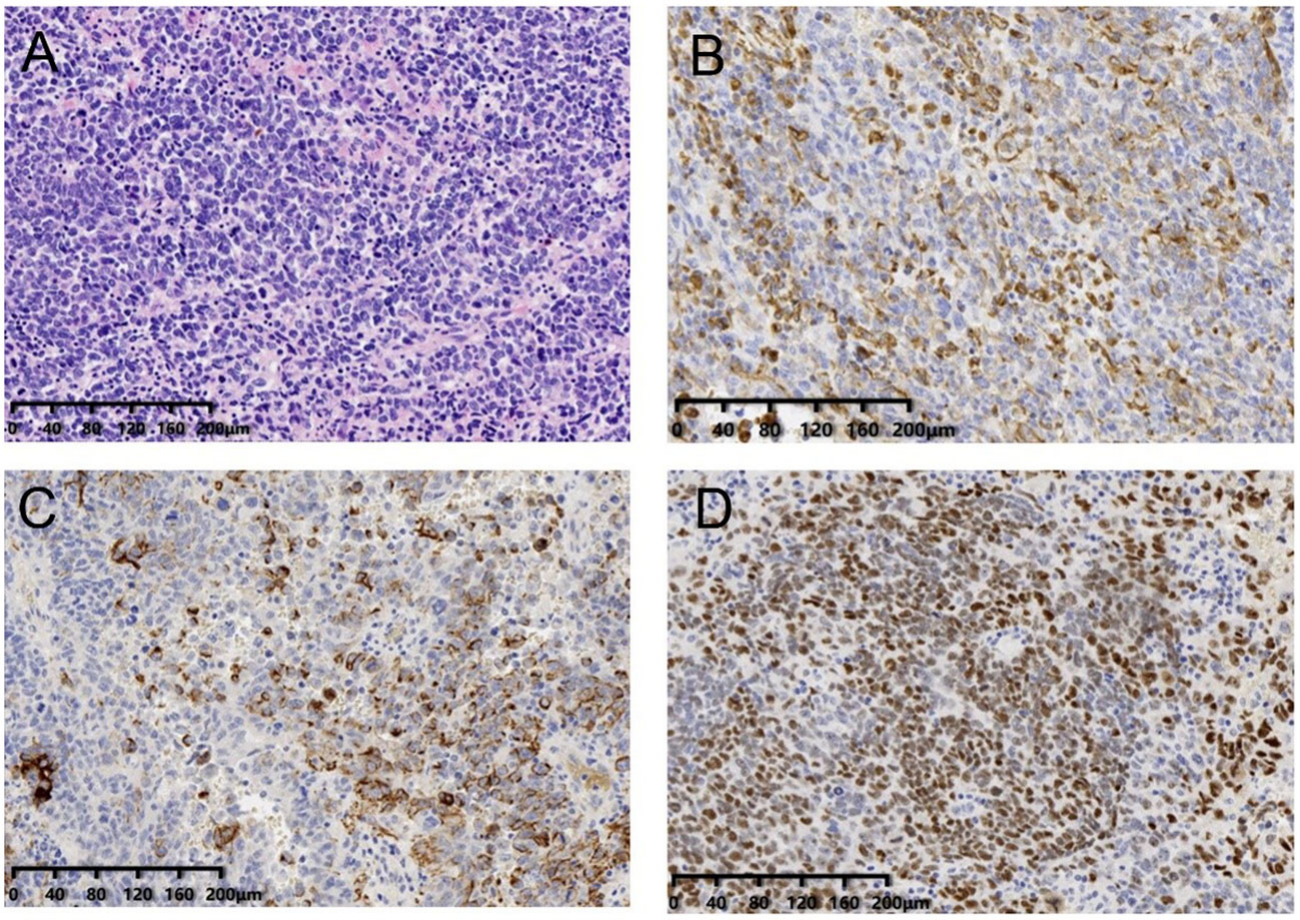
Immumohistochemical staining.

The patient recovered smoothly after operation, resumed exhaust on the 3rd postoperative day, and resumed a diet on the 5th day. The advanced age and the complexity of the surgery exposed the patient to postoperative electrolyte imbalance and low levels of albumin. Following the resumption of oral intake on the 5th day after surgery, continued observation was warranted. The patient was discharged from the hospital 15 days after the operation. The postoperative pathological findings revealed that the abdominal mass was a nodular mass with a size of 12.5×11×8cm. Under the microscope, the tumor cells showed solid aggregation, papillary and adenoid infiltration, moderate to severe atypia, common mitotic images, accompanied by flake necrosis, bleeding, and calcification. The IHC staining (**Figure 4**) results indicated the tumor as malignant epithelial one, considered as high-grade serous carcinoma, which might have originated from the female reproductive system or peritoneum. The tumor reached the muscular layer and was surrounded by multiple nodules of cancer in the fatty tissue of omental fibers.

The patient had no history of reproductive system diseases. The tumor markers of the patient at admission showed that CEA, CA19-9 and CA72-4 were all within the normal range, and no abnormal reproductive system was observed in preoperative imaging. To this end, the tumor was considered primary peritoneum. The tumor tissue of the patient was sequenced by panel 1238 and PD-L1. The results demonstrated five individual cell mutations, three of which were associated with targeted drugs. The tumor mutation load (TMB) was TMB-L, which indicated a low tumor mutation load. Besides, there was no corresponding immune drug that could benefit from it. The state of the microsatellite was MSS, suggesting the stability of the microsatellite, and no corresponding immune-related medication was recommended (**Figure 4**). Two positive immune-related genes, i.e., BRCA2 and TP53, were detected, and two germline mutations were detected, but both were non-pathogenic, and the possibility of inheritance was not high. The detection result of PD-L1 was CPS15, TPS3%. The patient is currently receiving carboplatin, paclitaxel combined with bevacizumab, and the therapeutic effect is still under observation.

So far, the patient has shown no signs of tumor recurrence and metastasis. Overall, this case highlights the importance of prompt diagnosis and treatment in patients suffering from abdominal distension, stopped defecation, abdominal pain, and hematemesis, as it may be a sign.

## Discussion

Primary peritoneal carcinoma (PPC) is a type of cancer originating from the primary peritoneal mesothelium organization, which causes diffuse cancerous tumors in the abdominal and pelvic regions. Though featuring a low incidence of PPC, it mainly affects middle-aged and elderly women (3) who often exhibit symptoms of abdominal distension, increased abdominal girth, and loss of appetite, terribly hindering the diagnosis in the early stages.

Muller’s system theory is considered the histological origin of PPC by most scholars. The female ovary and peritoneal epithelium originate from embryonic coelom epithelium and its inferior stroma, with the capacity to differentiate into Mullerian duct epithelium and its stroma. In cases of peritoneal cancer, the histological features and antigenicity of the tumor closely resemble those of female Mullerian duct tumors, and both can be identified through immunohistochemical methods.

The occurrence and development of peritoneal cancer have unique biological laws and characteristics that typically originate from the micro-metastasis of peritoneal free cancer cells (4). Two theories concerning the different sources of peritoneal free cancer cells have been developed, involving the tumor cell colonization theory and tumor cell encapsulation theory.

Pathologically, the most common types of PPC are serous papillary carcinoma, intrauterine carcinoma, transitional cell carcinoma, clear cell carcinoma, and malignant mixed Muller tube tumor. Serous adenocarcinoma is the most common form of primary peritoneal serous papillary carcinoma (PPSPC), a rare primary peritoneal tumor mainly found in elderly and postmenopausal women. PPSPC patients present with similar symptoms to those of primary ovarian serous papillary cancer (OSPC), such as abdominal pain, abdominal distension, ascites, intestinal obstruction, and significantly elevated serum CA125. Most patients have peritoneal implantation or metastasis at the time of visit, while 20% to 30% may suffer from regional lymph node metastasis (such as bilateral para-iliac and para-aortic) (5).

Their molecular similarity, clinical features, diffusion model, response to therapy, and survival rates make it challenging to distinguish histologically between PPSPC and epithelial ovarian cancer (EOC) (6).

Recent studies have reported that approximately 10% of ovarian cancer cases are associated with primary peritoneal carcinoma (PPC), with older women between the ages of 50 to 75 years old terribly affected. This is 8.3 years older than the age of onset for ovarian cancer during the same period (7). In this case, PPC should be necessarily considered a possible diagnosis in elderly women with bilateral appendages without abnormal pelvic cavity mass and ascites, especially when the ascites cancer cell test is positive and CA125 levels increase abnormally.

Diagnostic tests, such as color ultrasound or CT scans, can detect adnexal thickening or irregular thickening of the omentum, and in some patients, turbidity. However, postoperative histopathological examination and immunohistochemical staining stand as the gold standard for diagnosing PPC (8). Immunohistochemical staining also aids in the differential diagnosis of PPSPC, peritoneal malignant mesothelioma (PMM), primary epithelial ovarian cancer (PEOC), and gastrointestinal neoplasms peritoneal carcinoma (SPCGT) (7).

Studies have shown that the sensitivity and specificity of immunohistochemical markers such as CK7 and CA125 for diagnosing digestive or reproductive system tumors could be 100%. At least 2-3 positive immunobiomarkers, along with macroscopic features, can aid in diagnosis. For example, CK7 and P53 are positive markers, while CK20 is negative in PPSPC patients, which is consistent with literature reports (9).

Clinical and surgical characteristics of PPSPC patients are similar to those of phase III-IV ovarian papillary serous carcinoma (OPSC) ones. Treatment strategies for stage III-IV OPSC can also be applied to PPSCS, resulting in comparable or even better survival rates for PPSCS patients.

In summary, a thorough diagnostic workup, including histopathological examination and immunohistochemical staining, is necessarily important for the accurate diagnosis of PPC. Moreover, treatment strategies for advanced OPSC can also be applied to PPSCS, resulting in comparable survival rates.

Peritoneal cancer, also known as primary peritoneal carcinoma (PPC), is a rare form of cancer that affects the peritoneum, which is the lining of the abdominal cavity. The treatment of PPC is complex and often requires a multi-disciplinary approach, involving surgery, chemotherapy, and radiation therapy.

Meanwhile, it has been observed that extensive tumor resection alone is not sufficient for the treatment of PPC, which, if not combined with abdominal chemotherapy, can lead to massive peritoneal implantation, thereby failing to prolong the survival of patients and seriously affecting the quality of life. In this case, surgeons are required to be proficient in targeted chemotherapy techniques when treating peritoneal cancer patients.

The current treatment principle for PPC and advanced ovarian cancer is basically the same, with the core being cytoreductive surgery (CRS) and hyperthermic intraperitoneal chemotherapy (HIPEC). The basic principle of this treatment system is to resect the macroscopic lesions by CRS and treat the microscopic lesions to achieve radical treatment at the histological level. Micro-metastases and free cancer cells in the abdomen and pelvis are eliminated using HIPEC to achieve cytological radical treatment.

CRS combined with preoperative/postoperative platinum-based chemotherapy may be effective in PPSPC patients. The spread of primary peritoneal carcinoma is often confined to the abdominal cavity and accompanied by ascites. The peritoneum and lower diaphragmatic surface are widely planted but rarely penetrate the peritoneum. This unique biological feature forges a foundation for intraperitoneal drug use.

The adoption of preoperative intraperitoneal chemotherapy has emerged as a novel option, with surgical resection undertaken after localizing the lesion. Hyperthermic intraperitoneal chemotherapy for peritoneal cancer management offers both open and closed methods, providing flexibility in choosing from various treatment regimens such as the Sugarbaker regimen, oxaliplatin regimen, incorporating 5-FU, mitomycin C, cisplatin, and other agents. Tailored treatment approaches should be determined in accordance with the most current clinical guidelines. Studies have shown that preoperative chemotherapy for PPC is provided with several advantages. First, the drug concentration of intraperitoneal chemotherapy can be 10 ~ 100 times that of intravenous chemotherapy, which means that chemotherapy drugs directly kill tumor cells, with a high local control rate. Second, preoperative intraperitoneal chemotherapy can degrade the disease, reduce the scope of surgery, and improve the success rate of CRS. Moreover, surgical trauma is reduced, thereby providing better conditions for postoperative chemotherapy. Finally, the dose of intravenous chemotherapy can be reduced, the adverse reactions of systemic chemotherapy can be significantly reduced, the physical condition of patients can be restored, and the tolerance of surgery and chemotherapy can be increased. At the same time, in preoperative chemotherapy, intraperitoneal chemotherapy is considered superior to intravenous chemotherapy in improving the efficacy of PPC.

In conclusion, the treatment of PPC requires a comprehensive approach, and targeted chemotherapy techniques must be used by proficient surgeons. The current treatment principle of PPC and advanced ovarian cancer is based on CRS and HIPEC. Preoperative intraperitoneal chemotherapy has become a new option, which presents several advantages, including a high local control rate, the chance to degrade the disease, and reduced surgical trauma, effectively facilitating postoperative chemotherapy.

In the treatment of peritoneal carcinomatosis (PPC), the potential for peritoneal implantation during tumor resection should be necessarily taken into account, which can negatively impact patient survival and quality of life. To address this challenge, targeted chemotherapy techniques must be employed by proficient surgeons. The current treatment principle for PPC and advanced ovarian cancer involves cytoreductive surgery (CRS) and hyperthermic intraperitoneal chemotherapy (HIPEC), which work jointly to resect macroscopic lesions and treat them at the histological and cytological levels.

Studies have shown that combining preoperative or postoperative platinum-based chemotherapy with CRS can be effective in treating PPC patients. Due to the unique biological features of PPC, intraperitoneal chemotherapy has proven to be particularly effective, with a drug concentration up to 100 times higher than intravenous chemotherapy. Preoperative intraperitoneal chemotherapy is also endowed with the advantages of reducing the scope of surgery, improving the success rate of CRS, and reducing adverse reactions of systemic chemotherapy.

For patients with a large amount of ascites, concurrent intraperitoneal chemotherapy with cisplatin has been found to be more effective in controlling ascites and residual tumor lesions than postoperative residual lesions (10). However, early chemotherapy can interfere with CRS and prolong the recovery time of the patient, and is thus not recommended. To minimize trauma and improve postoperative chemotherapy and prognosis, intraoperative tumor focus should be reduced, and the surgery should be staged accordingly.

In conclusion, a combination of targeted chemotherapy techniques and cytoreductive surgery is the current standard of care for treating PPC and advanced ovarian cancer. By tailoring treatment to the unique biological characteristics of PPC, such as utilizing intraperitoneal chemotherapy and minimizing surgical trauma, patients are allowed to achieve better outcomes and quality of life.

PPC, a rare form of cancer, originates in the peritoneum, the abdominal lining, and shares notable similarities with ovarian cancer, encompassing symptoms and treatment modalities. Recent research has indicated a potential common origin for PPC and ovarian cancer, suggesting that both may arise from the same type of cells.

However, despite the similarities, there are also some key differences between PPC and ovarian cancer. For example, the prognosis for PPC is generally worse than that of concurrent ovarian cancer. Some scholars have suggested that there may be no significant difference in prognosis between the two (11), but the research conducted by Nam and others shows that the overall survival time for PPC is 41 months, with an overall 5-year survival rate of merely 18.1% (12).

In effect, there are several factors that may influence the prognosis of PPC. Of them, age is one important factor, with older patients generally subject to a poorer prognosis. The size of the residual lesion after surgery is also a significant prognostic factor, as larger lesions are more difficult to remove completely. Pathological grade, or the degree of malignancy of the cancer cells, is another crucial factor, with higher-grade tumors generally presenting a worse prognosis.

The duration of postoperative chemotherapy and the sensitivity of the patient to chemotherapy drugs may also affect the prognosis of PPC. Patients who respond well to chemotherapy and receive a longer course of treatment may have a better prognosis than those who do not.

Despite the challenges posed by PPC, there is optimism for individuals diagnosed with this rare form of cancer. Early detection and appropriate treatment offer the potential for long-term remission or even a cure for some patients. The ongoing research dedicated to understanding the causes and refining treatment strategies for PPC is crucial in enhancing outcomes for individuals grappling with this complex disease.

## Data availability statement

The raw data supporting the conclusions of this article will be made available by the authors, without undue reservation.

## Ethics statement

Written informed consent was obtained from the individual(s) for the publication of any potentially identifiable images or data included in this article.

## Author contributions

YL: Investigation, Writing – original draft, Writing – review & editing. SW: Investigation, Writing – review & editing. YW: Investigation, Writing – review & editing. GC: Writing – review & editing. JZ: Investigation, Writing – review & editing. XW: Investigation, Writing – review & editing.
